# Genome edited sheep and cattle

**DOI:** 10.1007/s11248-014-9832-x

**Published:** 2014-09-10

**Authors:** Chris Proudfoot, Daniel F. Carlson, Rachel Huddart, Charles R. Long, Jane H. Pryor, Tim J. King, Simon G. Lillico, Alan J. Mileham, David G. McLaren, C. Bruce A. Whitelaw, Scott C. Fahrenkrug

**Affiliations:** 1 The Roslin Institute and R(D)SVS, University of Edinburgh, Easter Bush Campus, Edinburgh, EH25 9RG UK; 2 Recombinetics Inc, 2575 University Ave. West, Suite 100, Saint Paul, MN 55108 USA; 3 Department of Veterinary Physiology and Pharmacology, College of Veterinary Medicine, Texas A&M University, College Station, TX 77843 USA; 4 Genus plc, 1525 River Road, DeForest, WI 53532 USA

**Keywords:** Livestock, TALEN, Myostatin, Zygote, Genetic engineering

## Abstract

Genome editing tools enable efficient and accurate genome manipulation. An enhanced ability to modify the genomes of livestock species could be utilized to improve disease resistance, productivity or breeding capability as well as the generation of new biomedical models. To date, with respect to the direct injection of genome editor mRNA into livestock zygotes, this technology has been limited to the generation of pigs with edited genomes. To capture the far-reaching applications of gene-editing, from disease modelling to agricultural improvement, the technology must be easily applied to a number of species using a variety of approaches. In this study, we demonstrate zygote injection of TALEN mRNA can also produce gene-edited cattle and sheep. In both species we have targeted the myostatin (*MSTN*) gene. In addition, we report a critical innovation for application of gene-editing to the cattle industry whereby gene-edited calves can be produced with specified genetics by ovum pickup, in vitro fertilization and zygote microinjection (OPU-IVF-ZM). This provides a practical alternative to somatic cell nuclear transfer for gene knockout or introgression of desirable alleles into a target breed/genetic line.

## Introduction

The ability to generate gene knockouts is an extremely powerful tool for the analysis of gene function and for the generation of animals with biotechnological or breeding applications (Fahrenkrug et al. 2010). In livestock species this process traditionally involves the generation of a knock-out cell line generated utilising homologous recombination followed by somatic cell nuclear transfer (SCNT). This remains the method of choice for many applications (Kurome et al. 2013), however, application of SCNT strategies requires a high-level of technical expertise, reliable supply of oocytes and a large recipient herd, features not available in many areas where gene-editing might have the greatest impact.

The advent of highly efficient genome editors has driven a renaissance in livestock genetic modification by embryo microinjection (Tan et al. 2012; Lillico et al. 2013). Whereas pronuclear injection, the original method for creation of transgenic livestock, is rarely performed nowadays due to the low frequency of transgenic offspring (Clark and Whitelaw 2003; Clark et al. 2007; Ivics et al. 2014) cloning strategies are still widely utilised and in combination with zinc finger nucleases have been used to generate edited cattle (Liu et al. 2014), pigs (Hauschild et al. 2011), sheep (Zhang et al. 2014) and goats (Boulanger et al. 2014). In comparison to cloning, cytoplasmic injection of zygotes with editor mRNA is both technically simple and efficient (Geurts et al. 2009; Carbery et al. 2010; Carlson et al. 2012). In this study, we build on our recent success of gene-editing in pigs (Lillico et al. 2013) to derive the first genome edited sheep and cattle. As with our swine study, the editing events were the result of direct injection of TALEN mRNA into zygotes followed by transfer into synchronized recipients. Another critical innovation in this study is that bovine embryos were prepared by ovum pickup, in vitro fertilisation and zygote microinjection (OPU-IVF-ZM). OPU-IVF is widely used in the cattle industry to rapidly produce a number of offspring from a single cow of elite genetics, up to 50–100 offspring per year (Leeuw 2006). Thus, in vitro produced embryos from either in vitro or in vivo matured oocytes can be used for rapid introgression of gene-edits into defined populations.

## Materials and methods

### TALEN design and construction

Design and construction of the btGDF83.1L+83.1NR is described in Carlson et al. 2012 using the RCIscript-GoldyTALEN transcription vector (Addgene ID 38142). Messenger RNA was synthesized using the mMessage Machine T3 Kit (Ambion) as previously described (Carlson et al. 2012) prior to polyadenylation using the Poly(A) tailing kit (Ambion) according to the manufacturers recommendations. To test the activity of the TALEN pair, 1 µg of mRNA was transfected (Neon™, Life Technologies; 1800 V, 20 ms, 1 pulse) into 10^6^ primary bovine and ovine fibroblasts. The cells were allowed to recover at 30 °C for 3 days before being harvested and the extent of genome modification assessed by Surveyor nuclease assay. The primer pair *MSTN* For (5′-GTCAAGGTAACAGACACACC-3′) and *MSTN* Rev (5′-CACCCACAGCGATCTACTAC-3′) was used to amplify a 359 base pair region both the bovine and ovine Surveyor assays.

### Bovine oocyte collection and manipulation

Oocytes were collected from Nellore cows under ultrasound guidance (Aloka 500 and a vaginal guide probe) with a 17 gauge needle connected via a Cook pump set at 72 psi. Oocytes were rinsed with pre-warmed TL Hepes (0.3 % BSA) + Gentamicin (50 µg/µl) supplemented with 10 IU/ml of Heparin and placed into maturation medium. In vitro fertilization was conducted in pre-equilibrated modified Tyrode-lactate medium supplemented with 250 µM sodium pyruvate, 1 % P/S, 6 mg/ml BSA Fatty Acid free (Sigma), 20 µM Penicillamine, 10 µM Hypotaurine, 1 µM Epinephrine and 10 µg/ml Heparin (Sigma) at 38.5 °C, 5 % CO_2_ in an air humidified incubator. Frozen semen from a Nelore bull was thawed at 35 °C then separated by centrifugation at 200×*g* in a density gradient medium (Isolate^®^, Irvine Scientific, Santa Ana, CA, USA) 50 % upper/90 % lower. The sperm pellet was re-suspended in 2 ml of modified Tyrodes medium and centrifuged at 200×*g* for 10 min to wash. This was repeated once more before the sperm pellet was removed and placed into a warm 0.65 ml microtube (VWR Scientific, Pittsburgh, PA., USA). Fertilization was conducted in a Nunclon^®^ 4-well multi-dish (VWR) containing up to 50 matured oocytes per well and a concentration of 1.5 × 10^6^ sperm/ml + 20 µg/ml heparin. Presumptive zygotes, 20–22 h post fertilization, were vortexed and further cleaned with a stripper pipette (125 µm diameter) prior to placing in Hanks 199 + 10 % FBS + Gentamicin for an injection of either 2 or 5 ng/µl TALEN mRNA. Injections were conducted under positive pressure until a slight expansion of the cell membrane was observed. All injected zygotes were placed in Evolve + 4 mg/ml Probumin (BSA) + Gentamicin in 5/5/90 humidified incubator at 38.5 °C. On day 2 post IVF, all non-cleaved embryos were removed leaving the remainder to culture undisturbed until day 7. At 7 days post IVF viable embryos were washed through Vigro Holding Medium (Bioniche), loaded and transferred into synchronized cross-bred recipients.

### Ovine oocyte manipulation and transfer

Ovine ovaries were collected from the abattoir and the follicles aspirated with pre-warmed phosphate buffered saline at 38 °C. Oocytes were washed three times in oocyte wash medium before transfer to maturation medium for 22 h (38.5 °C, 5 % CO_2_). The oocytes are then washed twice in fertilisation medium before being transferred to a Nunclon^®^ 4-well multi-dish (VWR) with each well containing 450 ul of fertilisation medium and approximately 40 oocytes. 1 × 10^6^ sperm was added to each well and incubated for 24 h (38.5 °C, 5 % CO_2_). The fertilized oocytes were then washed in SOFaaBSA to remove sperm and any remaining cumulus cells. The zygotes were subjected to a single 2–5pl injection of 2 ng/ul TALEN mRNA before being returned to 4 well plates containing 800 ul SOFaaBSA medium per well and cultured in groups of approximately 40 zygotes. The zygotes were incubated (5 % CO_2_, 5 % O_2_, 90 % N_2_, 38.5 °C) for 6–7 days at which point blastocysts were transferred to recipient ewes. Progesterone sponges (Chronogest sponges) were inserted into ewes for a period of between 11 and 15 days. After removal of the sponges the ewes showed estrus 36–48 h later. Schedules were arranged such that day 6 blastocysts were transferred to recipient ewes 6 days post estrus under general anaesthesia, following a mid-line laparotomy to expose the uterus. A Drummond pipette was used to transfer two or three blastocysts into the uterine horn.

### Genotyping

Gene editing events were characterized by PCR, Surveyor assays and sequencing as described previously (Carlson et al. 2012). Analysis of bovine samples utilized the primer pair btGDF8 forward (5′-CCTTGAGGTAGGAGAGTGTTTTGGG-3′) and btGDF8 reverse (5′-CTCATGAACACCCACAGCGATCTAC-3′). The lambs were analysed using the primer pair *MSTN* For (5′-GTCAAGGTAACAGACACACC-3′) and *MSTN* Rev (5′-CACCCACAGCGATCTACTAC-3′).

## Results and discussion

The aim of this paper was to determine the potential of genome editors as a tool for introducing desired mutations in sheep and cattle species. The *MSTN* gene (McPherron et al. 1997) was considered an attractive target as mutations have been found naturally in both cattle (Grobet et al. 1997) and sheep (Clop et al. 2006). *MSTN* or growth and differentiation factor 8 (GDF-8) is a member of the transforming growth factor β family and is a negative regulator of muscle growth. An 11 bp deletion in the bovine *MSTN* gene at position 821 has been shown to result in muscular hypertrophy or the ‘double muscle’ phenotype characterised by a 20 % increase in muscle mass (Grobet et al. 1997). Mutations in the *MSTN* gene that result in either inactivation or reduction of functional protein also result in a marked increase in muscle mass (McPherron and Lee 1997). Indel formation induced by TALEN activity would be ideal for attempting to replicate the double muscle phenotype and proving the functionality or editors in sheep and cattle. In this particular example, we hypothesized that the easy calving of Nelore cattle will reduce the management burden of dystocia that is common in other cattle breeds with double muscling breeds (Ménissier 1976). For sheep, higher survivability of offspring derived from a terminal Texel (*MSTN*-KO) sire (Leymaster and Jenkins 1993) would be more beneficial in alternative breeds of sheep. Gene editing represents a rapid methodology to introgress *MSTN* inactivating alleles into naive breeds.

**Fig. 1:**
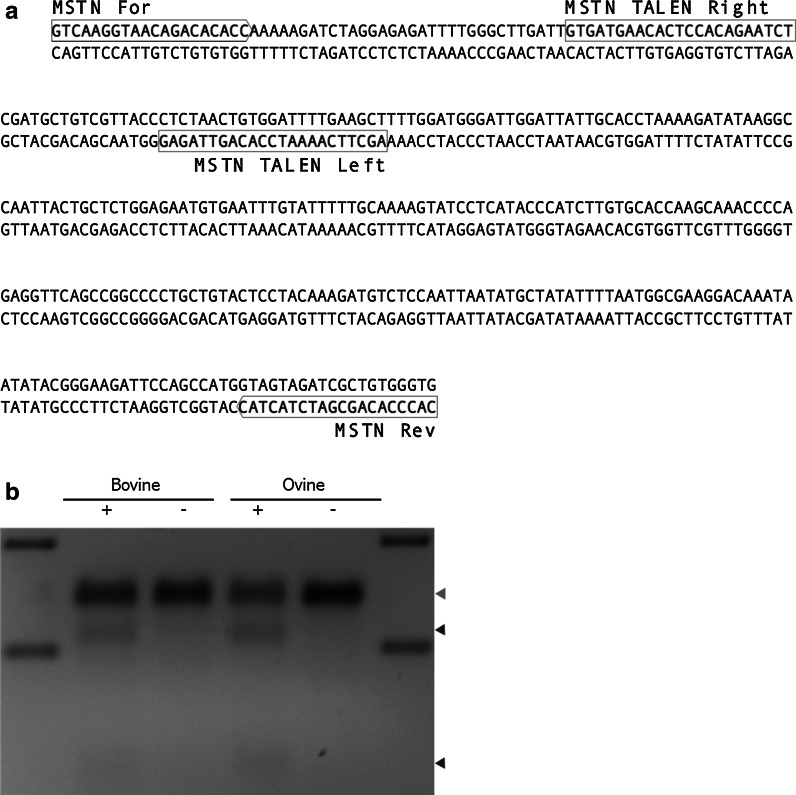
The *MSTN* TALENs. **a** 359 bp of the bovine *MSTN* gene sequence showing the TALEN binding sites (*red boxes*) and the primers (*green boxes*) used to amplify the region for the surveyor nuclease assay. The base coloured *blue* dictates the position of the mismatch in the ovine sequence in which it is G rather than A. **b** The surveyor nuclease assay results for the TALEN transfected bovine and ovine fibroblasts. gDNA extracted from transfected cells was treated with and without nuclease. (Color figure online)

We have previously demonstrated activity of TALENs that targeted a region of the bovine *MSTN* gene between positions 815 and 872 relative to the start codon (NM_001001525; Fig. 1a) (Carlson et al. 2012). The left TALEN recognises 23 base pairs and the right TALEN recognises 19 base pairs. Comparison of the bovine and ovine (NM_001009428) sequences showed there to be a SNP at base 8 of the binding site of the left TALEN monomer (Fig. 1a). We have observed that at least one mismatch between the TALENs and the target sequence can be tolerated (Tan et al. 2013), so we hypothesized that the bovine TALENs would also have activity in ovine cells. Indeed, transient transfection of TALEN mRNA into bovine and ovine fibroblasts and subsequent Surveyor nuclease assay showed similar levels of activity in both species (Fig. 1b).

### Bovine zygote injections and transfers

**Fig. 2:**
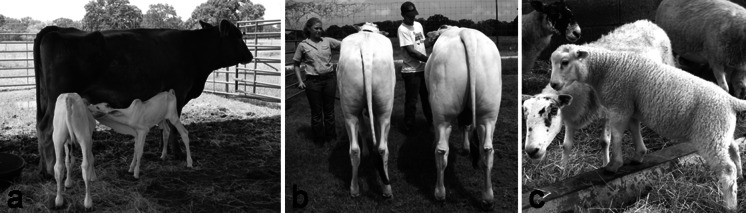
*MSTN* edited animals. **a** The live born bull (bull #1: *left*) and heifer calf (*right*). **b** The readily observed phenotypic difference between bull #1 (*right*) and the wild-type heifer (*left*). **c** The edited lamb

**Fig. 3:**
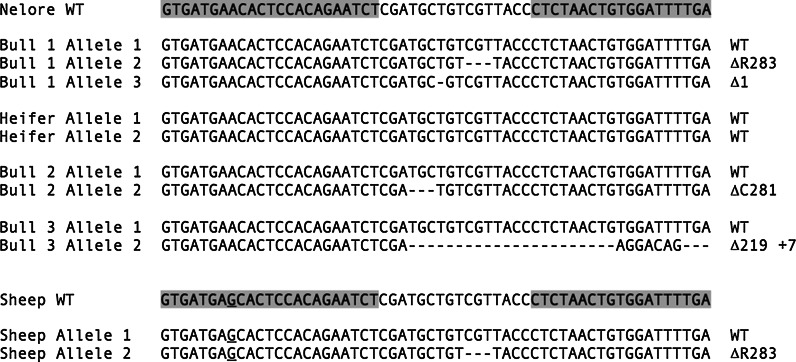
The *MSTN* editing events. An alignment of the bovine and ovine WT sequences and the alleles present in each of the edited animals. The TALEN binding sites are highlighted on the WT sequences, the ovine mismatch is *underlined* and the corresponding amino acid change is indicated on the *right*

Two rounds of OPU-IVF were conducted using Nelore donors in parallel to the same procedure with abattoir oocytes (TransOva Genetics, Sioux Center, IA). After IVF, presumptive zygotes were injected with 2 or 5 ng/µl of TALEN mRNA. Embryos were scored for blastocyst formation on day 7 and a portion of embryos were analysed for gene-edits to evaluate performance of the approach. For embryos derived from abattoir oocytes, 4/13 (31 %) and 17/30 (57 %) were edited for the 2 and 5 ng/µl injections, respectively. Four of the six Nelore blastocysts included in this analysis were edited. In total, 20 Nelore embryos were transferred to 11 outbred recipients resulting in two full term twin-pregnancies (Table 1). The first set of twins birthed naturally resulting in a bull (bull #1) and heifer calf (Fig. 2a). Unfortunately, the second recipient went into labour shortly after a routine check and the birth was unattended. Both bull calves were born dead due to calving difficulties associated with twinning. Sequence analysis revealed that each of the three bull calves was edited whereas no edits were observed in the heifer. A total of 3 unique alleles were sequenced from bull # 1. The predominant genotype 844del1 (70 % of sequenced alleles, n = 13) is a frame shift mutation that results in a stop codon within four amino acids. A second mutant allele, ΔR283, was also observed twice as was the wild type sequence (Fig. 3). This suggests that the TALENs were active for more than one cell division, an observation made previously by analysis of pre-implantation embryos (Carlson et al. 2012). The ΔR283 mutation has not been characterized previously, and since it is not a frame-shift it is expected to produce a protein, but with unknown functionality. Regardless, a phenotypic difference between bull #1 and the wild-type heifer is readily observed (Fig. 2b). Given that this bull is mosaic, without segregation it is difficult to differentiate whether hypermuscularity in the Nelore bull derives from haploinsufficiency, from homozygosity of 844del1 knockout allele, or heterozygosity 844del1 and ΔR283 alleles. The potential of ΔR283 as a hypomorphic allele will be evaluated in subsequent generations due to the desire to identify myostatin genotypes that balance enhanced muscle hypertrophy with calving ease (Keele and Fahrenkrug 2001). Future analysis will also measure the effect of the mosaicism on germline transmission of the editing events from the Nelore bull. Given this, a full analysis of the degree of mosaicism in different tissues, to assess whether or not it is lower in muscle, has been ruled out at this stage.

**Table 1:** The development and editing frequency of bovine and ovine zygotes

Dose	Species	Oocytes	Cleaved (%)	Blastocysts (%)	Transferred	Preg (%)	Edited (%)
C	Transova	83	62 (74)	27 (33)	–		
TE	Transova	119	89 (75)	24 (20)	–		
2 ng/µl	Transova	45	34 (76)	12 (27)	–		
	Nelore	21	17 (81)	6 (29)	4	0/2	–
	Sheep	113	61 (54)	27 (24)	26	8/9 (89)	1/9 (11)
5 ng/µl	Transova	308	234 (76)	44 (14)	–		
	Nelore	166	112 (67)	13 (8)	16^a^	2/9 (22)	3/4 (75)

A comparison of the zygote data, pregnancy rates and editing frequencies

*C* uninjected controls, *TE* TE injected controls

^a^9 of the transferred were morulae

### Ovine zygote injections and transfers

Ovine oocytes were collected from abattoir-derived material and subjected to in vitro maturation and in vitro fertilisation (Ritchie et al. 2008) before receiving a single 2–5pl injection of TALEN mRNA at 2 ng/µl. The zygotes were subsequently cultured for a further 6–7 days before transfer of blastocysts to synchronised recipient ewes. The sheep zygotes showed a good blastocyst development rate of 24 %, despite an initially poorer than expected cleavage rate (Table 1). In total 26 blastocysts were transferred to 9 recipient ewes (2 or 3 blastocysts per ewe) resulting in 8 pregnancies and 12 live births. Three of these lambs died within 24 h post-partum and carcasses were disposed of before samples could be acquired for analysis.

Of the 9 live births one was shown to be edited (Fig. 2c) as a heterozygote ∆R283 (Fig. 3), demonstrating cross-species application and surprisingly an identical genotypic outcome of TALENs designed against the bovine myostatin gene. As with bull #1, the sequence context does not enable definitive identification as to which three bases have been deleted from the MSTN gene. For example, in Fig. 3, the three deleted bases have been marked as those coding for R283, alternatively, the three-base deletion could equally have started 1 or 2 bases downstream. However, in all three scenarios the resulting nucleotide and amino acid sequences would be the same.

## Conclusion

This study further exemplifies the utility and ease with which TALENs can be used to engineer the genome of livestock. Specifically we demonstrate that sheep and cattle can be added to the growing list of species for which genome editing is now practical. It is anticipated that these tools will accelerate the utilisation of engineered livestock for biomedical and agricultural applications. Genome edited livestock differ from traditional GM animals in that no recombinant DNA (transgene) is integrated into the animal genome. Combined with the ability to mimic desirable or pre-existing mutations, genome editing overcomes many of the issues associated GM animals increasing the likelihood for societal acceptance. Furthermore, the advent of this technology is extremely timely given the global challenge of food security; targeted mutagenesis and allele introgression has the potential to accelerate genetic advancement of agriculturally important traits. The deployment of gene editing using industry-standard reproductive technologies demonstrates several practical approaches to advancing livestock genetics and biotechnology.
